# Bactericera cockerelli Picorna-like Virus and Three New Viruses Found Circulating in Populations of Potato/Tomato Psyllids (*Bactericera cockerelli*)

**DOI:** 10.3390/v16030415

**Published:** 2024-03-08

**Authors:** Jennifer Dahan, Gardenia E. Orellana, Kaleigh B. Wald, Erik J. Wenninger, W. Rodney Cooper, Alexander V. Karasev

**Affiliations:** 1 Department of Entomology, Plant Pathology, and Nematology, University of Idaho, 875 Perimeter Drive, MS 2329, Moscow, ID 83844-2329, USA; jdahan@uidaho.edu (J.D.); gardeniao@uidaho.edu (G.E.O.); wald9609@vandals.uidaho.edu (K.B.W.); 2 Department of Entomology, Plant Pathology, and Nematology, and Kimberly Research and Extension Center, University of Idaho, Kimberly, ID 83341-5082, USA; erikw@uidaho.edu; 3 USDA-ARS, Temperate Tree Fruit and Vegetable Research Unit, Wapato, WA 98951, USA; rodney.cooper@usda.gov

**Keywords:** ‘*Canditatus* Liberibacter solanacearum’, zebra chip disease, psyllid viruses

## Abstract

An investigation of viruses circulating in populations of field and laboratory potato/tomato psyllids (*Bactericera cockerelli*) was conducted using high-throughput sequencing (HTS) technology and conventional RT-PCR. Three new viruses were discovered: one from the family *Tymoviridae* and two from the family *Solemoviridae*. A tymo-like virus sequence represented a nearly complete 6843 nt genome of a virus named Bactericera cockerelli tymo-like virus (BcTLV) that spanned five open reading frames (ORFs) which encoded RNA-dependent RNA polymerase (RdRP), helicase, protease, methyltransferase, and a capsid protein. Phylogenetic analyses placed the RdRP of BcTLV inside a divergent lineage of the viruses from the family *Tymoviridae* found in insect and plant hosts in a sister clade to the genera *Tymovirus*, *Marafivirus*, and *Maculavirus*. Four solemo-like virus sequences were identified in the HTS outputs, representing two new viruses. One virus found only in field-collected psyllids and named Bactericera cockerelli solemo-like virus 1 (BcSLV-1) had a 5479 nt genome which spanned four ORFs encoding protease and RdRP. Three solemo-like sequences displayed 87.4–99.7% nucleotide sequence identity among themselves, representing variants or strains of the same virus named Bactericera cockerelli solemo-like virus 2 (BcSLV-2). The genome of BcSLV-2 spanned only two ORFs that encoded a protease and an RdRP. Phylogenetic analysis placed the RdRPs of BcSLV-1 and BcSLV-2 in two separate lineages as sister clades to viruses from the genus *Sobemovirus* found in plant hosts. All three new psyllid viruses were found circulating in psyllids collected from potato fields in southern Idaho along with a previously identified Bactericera cockerelli picorna-like virus. Any possible role of the three viruses in controlling populations of the field psyllids remains to be elucidated.

## 1. Introduction

*Bactericera cockerelli* (Hemiptera: Triozidae), the potato/tomato psyllid (PoP), primarily develops and reproduces on species within the Solanaceae family [1,2,3,4,5,6]. PoP transmits the pathogenic bacterium ‘*Candidatus* Liberibacter solanacearum’ (Lso), associated with the zebra chip (ZC) disease in potato, a significant threat to potato production [7]. While investigating the possible use of viruses as a potential future management strategy for ZC, we recently identified a new virus from PoP, Bactericera cockerelli picorna-like virus (BcPLV; [8]). BcPLV has a positive-sense RNA genome of about 10 kb with an organization resembling viruses from the family *Iflaviridae* [8]. BcPLV was initially identified in a publicly available transcriptomics dataset and later found circulating in two psyllid colonies maintained in the USDA research facility in Wapato, WA [8]. However, the circulation of BcPLV in field populations of PoP was not addressed at the time, and a systematic high-throughput sequencing (HTS) survey of PoP for the presence of viruses circulating in the psyllid was not conducted. In a related pathosystem, the Asian citrus psyllid *Diaphorina citri* (Hemiptera: Liviidae), vectoring another Liberibacter species, ‘*Candidatus* Liberibacter asiaticus’, responsible for the citrus disease Huanglongbing (HLB; [9,10,11]), was found to host several viruses that were suggested as potential biocontrol agents for managing psyllid populations [12,13,14,15,16]. Viruses found in Asian citrus psyllids belong to the families *Parvoviridae*, *Reoviridae*, *Flaviviridae*, and *Iflaviridae* [12,13,14,16,17]. In another related pathosystem, the wild lime psyllid (*Leuronota fagarae* Burckhardt) was found to host Leuronota fagarae picorna-like virus (LfPLV), which is closely related to BcPLV [18]. Up until now, no viruses from the families *Tymoviridae* and *Solemoviridae* have been reported in psyllids.

Viruses classified as tymovirids have isometrical particles of approximately 30 nm in diameter built of a single capsid protein (CP), and a single-stranded, positive-sense RNA genome of 6.0–7.5 kb in length [19]. The family *Tymoviridae* comprises three currently recognized genera infecting plants (*Tymovirus*, *Maculavirus*, and *Marafivirus*), as well as a number of unassigned members [19,20]. The genomes of tymovirids have an m^7^G-cap at the 5′ terminus, and the structure of the 3′ terminus may vary, with some being polyadenylated in marafi- and maculaviruses and non-polyadenylated in tymoviruses [19]. Some tymovirids are known to be vectored by arthropods and are able to replicate within those vectors [21]. Although the overwhelming majority of tymovirids have been found in plant hosts, tymo-like virus sequences have also been found in fungi and in insects [22,23,24,25]. Viruses classified in the family *Solemoviridae* have non-enveloped isometric particles of 20–34 nm in diameter and a single-stranded, positive-sense, non-polyadenylated RNA genome of 4–6 kb, which has a VPg protein at the 5′ end [26]. The family *Solemoviridae* comprises four genera: *Sobemovirus*, *Polerovirus*, *Enamovirus*, and *Polemovirus* [26]. The majority of solemovirids infect plant hosts, and members of the genera *Polerovirus* and *Enamovirus* are transmitted by insect vectors in a persistent, non-propagative manner [26]. Nevertheless, recently, two reports described solemo-like virus sequences identified in ticks [27] and in Odonata insects [28].

To address the knowledge gap related to viruses circulating in potato psyllid populations and to examine the range of viruses associated with PoP in a more systematic way, psyllid colonies maintained in our research facility were subjected to HTS analysis. For over a decade, the University of Idaho has maintained a potato psyllid monitoring program (from 2012 to 2023) collecting psyllids from potato fields in southern Idaho for subsequent Lso testing and psyllid haplotyping [29,30,31,32,33]. The second goal of our study was to test these field-collected psyllids for specific viruses by RT-PCR to assess virus prevalence and circulation in field populations. Here, we report three new positive-strand RNA viruses identified in psyllid colonies and in psyllids collected from potato fields.

## 2. Materials and Methods

### 2.1. Psyllid and Plant Sampling and Sample Processing

The laboratory psyllid colonies were maintained at the USDA-ARS greenhouse facility on potato cultivar ‘Ranger Russet’ and tomato cultivar ‘Moneymaker’ under controlled conditions (25 °C; 16:8 h day/night photoperiod). The colonies were established from psyllids that were originally collected in Texas in 2010 (PSC, central haplotype), Washington State in 2019 (PSW, western haplotype) and 2020–2021 (PSNW, northwestern haplotype). The colonies were periodically checked for the presence or absence of Lso [34] and *Wolbachia* (present in the western and central haplotypes only) [35], using diagnostic PCR, and for haplotype purity, based on sequences of the cytochrome oxidase 1 mitochondrial gene (CO1) [36]. For the HTS analyses, psyllids were collected in February 2021, from each of the three colonies, PSW, PSNW, and PSC, as described previously [8], and kept frozen at −80 °C. Psyllids from a potato field located near Kimberly, ID (KPF), were collected in August 2020, using the beat sheet method, and kept frozen at −80 °C.

As part of a potato psyllid/ZC risk assessment monitoring program in southern Idaho, yellow sticky traps were deployed and retrieved weekly in commercial potato fields [32]. Briefly, individual psyllids were visually identified, removed from traps, and tested for the presence of Lso, as described previously [29,30]. From July to September 2023, 218 potato psyllids were collected in this monitoring program and tested for the presence of BcPLV, BcTLV, BcSLV-1, and BcSLV-2 using RT-PCR.

### 2.2. RNA Extraction and HTS Analysis

For each sample, RNA was extracted from ten pooled psyllids. Insects were ground using a mortar and pestle in liquid nitrogen, and total RNA was extracted using the Trizol reagent following manufacturer’s instructions. Resulting RNAs were subjected to DNase treatment using the DNAse Max enzyme (Qiagen, Germantown, MD, USA), and subsequently concentrated using the RNA clean and concentration kit—5 (ZymoResearch, Orange, CA, USA). Ribosomal RNAs were then depleted using the RiboMinus Plant ribodepletion kit (ThermoFisher Scientific, Waltham, MA, USA) and resulting ribodepleted RNAs were concentrated by ethanol precipitation. Between 390 and 638 ng of ribodepleted RNA per sample was then subjected to library preparation and sequencing in paired-end 250 bp read format on a NovaSeq platform (Genomics and Bioinformatics Resources Core, IIDS, University of Idaho). 

Resulting paired-end reads were cleaned using Trimmomatic v0.38 in ILLUMINACLIP mode with the following settings: 2:30:10:8:TRUE LEADING:3 TRAILING:3 SLIDINGWINDOW:4:20 MINLEN:50. Paired, trimmed reads were assembled using SPAdes v3.15.3 in rna mode. Resulting contigs were then submitted to a BLASTn search against the nt database, with a cut-off e-value of 0.00001 and a max_target_seqs setting of 1. Contigs with no hits were then submitted to DIAMOND [37] v2.0.14 with BLASTx settings run locally (nr database as of February 2022) in –iterate mode with a max_target_seqs setting of 1, and all contigs with viral hits were then extracted and analyzed further in Geneious Prime 2021 (Biomatters, Inc., Boston, MA, USA). Mapping of clean reads against viral contigs was performed using Bowtie2 in the end-to-end mode, default settings [38].

### 2.3. Nucleic Acid Extraction, RT-PCR Testing, and Sanger Sequencing

For tomato, potato, and insect samples collected in February and April 2022, total nucleic acids were extracted according to the protocol of Dellaporta et al. [39] as modified in Dahan et al. [40]. Reverse transcription was performed using 4.5 µL of the total nucleic acid extract in a 25 µL reaction mixture that contained 5× first-strand buffer (Promega, Madison, WI, USA), 2.5 mM dNTP, 3 µM oligo dT + random hexamers, rRNasin Ribonuclease Inhibitor (Promega), and M-MLV reverse transcriptase (Promega). Before the reverse transcription reaction, the RNA template was incubated at 70 °C for 5 min, then the reverse transcription mix was added. The profile used consisted of incubation at 37 °C for 60 min and reverse transcriptase deactivation at 70 °C for 10 min. All PCR reactions were accomplished by GreenTaq (GenScript, Piscataway, NJ, USA) in a 20 µL reaction mixture that contained 10× GreenTaq buffer, 2.5 mM dNTP, 5 µM of each forward primer and reverse primer, 1.5 units of GreenTaq, and 2 µL cDNA template. The PCR profile consisted of denaturing at 94 °C for 2 min, and 35 cycles of 94 °C for 30 s, 55–65 °C for 30 s (depending on the melting temperature of primers used), 72 °C for 1 to 2 min (depending on the fragment length amplified), followed by a final extension for 10 min at 72 °C. Primers used for this study are listed in Supplemental Table S1. For Sanger sequencing, PCR fragments were treated with ExosapIt (Affymetrix, Cleveland, OH, USA), and submitted for sequencing to Elim Biopharmaceuticals, Inc. (Hayward, CA, USA). 

For potato psyllids collected in southern Idaho in July–September 2023, as part of the monitoring program for psyllid and Lso prevalence in potato fields, RNAs were extracted from individual insects using TRIzol Kit (Thermo Fisher Scientific) according to the manufacturer’s protocol. Testing for the presence of BcPLV, BcTLV, BcSLV-1, and BcSLV-2 was conducted using RT-PCR with primers listed in the Supplementary Table S1. 

### 2.4. Sequence and Phylogenetic Analysis

Full-length replicase proteins encoded by the virus genomes of members of the families *Alphaflexiviridae*, *Betaflexiviridae*, *Gammaflexiviridae*, *Deltaflexiviridae*, and *Tymoviridae* were used to assess taxonomic position of BcTLV within the order *Tymovirales*; additionally, CP domains of members of the family *Tymoviridae* were used to assess the taxonomic position of BcTLV within this family. The replicase amino acid domain of RefSeq sequences of viruses from the order *Tymovirales* were retrieved from NCBI, along with additional, recently classified tymovirid sequences. Sequences of viruses from the orders *Martellivirales* and *Hepevirales* were included as an outgroup. The RdRP domain alignment provided by the ICTV for the family *Solemoviridae* [26] was used for the phylogenetic analysis of BcSLV-1 and BcSLV-2, with the addition of several sequences, MF141064 (Norway luteo-like virus 1), NC_032234 (Hubei sobemo-like virus 29), MK889164 (Hubei sobemo-like virus 1), and MN831440 (Gingko biloba sobemo-like virus). For each phylogenetic reconstruction, the amino acid sequences were aligned using MAFFT (g-insi mode or l-insi mode, default settings). Phylogeny inference was conducted based on these alignments using the IQ-tree 2 software [41], with ModelFinder for model selection [42], and SH-aLRT and UFboot for bootstrapping (1000 replicates; [43,44]) to build an unrooted maximum likelihood (ML) tree. The resulting tree was visualized and edited in R with the ggtree package, using the outgroup to root the tree [45]. 

## 3. Results

The HTS analysis conducted on potato psyllids belonging to three haplotypes (PSC, PSW, and PSNW) collected from three individual laboratory colonies and from a potato field in Kimberly, ID (KPF) (Table 1), produced between 19,214,695 and 29,796,445 paired-end 250 bp reads that assembled into 38,685 (PSC), 31,324 (PSNW), 28,623 (PSW), and 39,073 (KPF) contigs longer than 1000 nt. All four psyllid samples contained potato virus S (PVS) sequences that in three of them, PSC, PSNW, and KPF, could be assembled into a nearly complete PVS genome (Table 1) with 5595 (PSC), 7044 (PSNW), and 933 (KPF) reads mapped. Two of the samples, PSC and PSW, contained BcPLV sequences that were assembled into a complete virus genome (Table 1) with 58,441 (PSC) and 187,787 reads mapped. One sample, PSW, contained a few reads assembled into short, 1224–3729 nt contigs identified as being potato virus Y (PVY)-specific (Table 1), and another sample, PSC, contained few reads producing a short contig of 1378 nt identified as being Impatiens necrotic spot virus (INSV) segment L-specific (not shown). These PVS-, PVY-, and INSV-specific contigs may be assumed to represent contaminants present in the host plants on which the three psyllid colonies were maintained, and ingested by psyllids in the course of feeding. BcPLV, on the other hand, is a psyllid virus infecting two of the three colonies maintained in the Wapato facility [8].

### 3.1. Genome Analysis and Confirmation of the Presence of a New Tymo-like Virus in Psyllids

In addition to the well-known virus sequences identified in psyllids from the three colonies, a large 6843 nt contig (13,724 reads mapped) was revealed in the PSW sample which encoded five ORFs exceeding 100 codons. A BLASTn search through the GenBank database returned no similar nucleotide sequences, while the CD-Search program [46], available at the National Center for Biotechnology Information (Bethesda, MD, USA), identified the RdRP, MT, PRO, HEL, and CP domains of tymo- and tymo-like viruses in two of the three protein products encoded by ORFs 1 and 3 of the 6843 nt contig. Given the presence of the replication-associated and CP domains in the encoded proteins, this 6843 nt contig was assumed to represent a complete or nearly complete genome of a novel positive-strand RNA virus named “Bactericera cockerelli tymo-like virus” (BcTLV) and this nearly complete genome was subjected to a more thorough molecular and bioinformatics analysis. The sequence has been deposited into GenBank under accession number PP408654.

**Figure 1:**
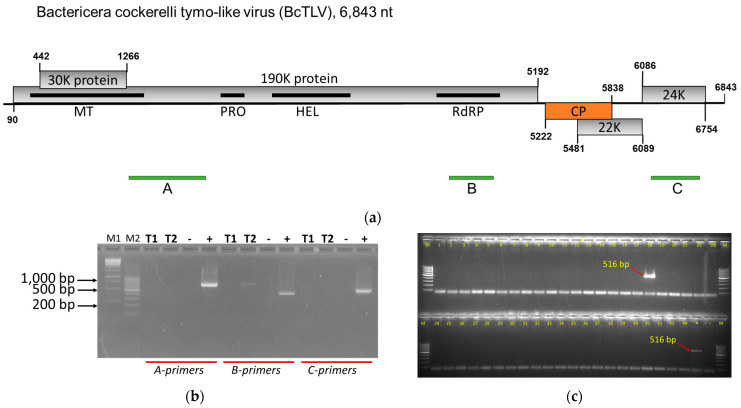
Bactericera cockerelli tymo-like virus (BcTLV) genome organization (**a**), its laboratory detection in potato psyllids and tomato plants (**b**), and the laboratory detection of BcTLV in field-collected insects (**c**). (**a**) Schematic diagram of the BcPLV genome with positions of the five open reading frames coding for the 30K protein, 190K protein, capsid protein (CP), 22K protein, and 24K protein shown as lightly shaded rectangles, with nucleotide numbers for the start and stop codons indicated. Black solid rectangles indicate positions of the known protein domains identified in the 190K protein: methyltransferase (MT), protease (PRO), helicase (HEL), and RNA-dependent RNA polymerase (RdRP). The green bars underneath the BcTLV genome represent the location of the RT-PCR fragments obtained using the primer pairs for testing (A, PSW_F1/R1, 702-bp; B, PSW_F2/R2, 421-bp; C, PSW_F3/R3, 516-bp). (**b**) Testing for BcTLV by endpoint RT-PCR in two tomato plants (T1 and T2) from the PSW colony of potato psyllids (*Bactericera cockerelli*), and in a bulked sample of ten *B. cockerelli* insects (+) collected in February 2021 and used for high-throughput sequencing; (–) negative non-template control. M1 and M2 are marker lanes, loaded with 1 kb (M1) and 100 bp (M2) DNA ladders. The sequences of the three sets of primers used for PCR amplifications (A, B, and C) are listed in Supplemental Table S1. (**c**) Testing for BcTLV by endpoint RT-PCR in 44 individual *B. cockerelli* insects collected in Canyon County of Idaho in August 2023; C-primers (Supplemental Table S1) were used for the testing here, producing a 516 bp PCR product; (+) positive control from the Wapato, WA, psyllid colony; (–) negative non-template control. M is a marker lane, loaded with the 100 bp DNA ladder.

The sequenced genome was conceptually translated and found to encode five open reading frames (ORFs), two of which were typical of tymo- and tymo-like viruses (Figure 1a). The largest, ORF1, spans positions 90 to 5192, encoding a 190 kDa polyprotein (190K protein) containing replication-related domains with presumed methytransferase (MT), protease (PRO), helicase (HEL), and RNA-dependent RNA polymerase (RdRP) activities. This 190K protein exhibited significant similarities with the polyproteins of tymo-, marafi-, and maculaviruses (up to 39% identity at the aa level), and also with tymo-like viruses found in insects (up to 48% identity) when subjected to the tBLASTn search through the GenBank databases. The smaller ORF2 completely overlaps with the 190K protein ORF1, starting at position 442 and ending at position 1266, encoding a 30 kDa protein (30K protein). This 30K protein seems to resemble similarly encoded products in genomes of other tymoviruses required for virus spread in a plant host and also functioning as gene silencing suppressors [47,48]; the sequence of this 30K protein did not exhibit significant similarities to the proteins in the GenBank databases though. The downstream ORF3 starts at position 5222 and ends at position 5838, encoding a 205 aa protein with an estimated molecular weight of 22.5 kDa (22.5K protein). This ORF3-encoded, 22.5K protein exhibited significant similarity with the CPs of tymo- and tymo-like capsid proteins (up to 30% identity) when compared to the GenBank databases using the tBLASTn program and was assumed to encode the CP. The downstream ORF4 partially overlaps with the CP-encoding ORF3, starting at position 5481 and ending at position 6089, encoding a 202 aa protein with an estimated molecular weight of 22.2 kDa (22K protein). The 3′ proximal ORF5 starts at position 6086 and ends at position 6754, encoding the 222 aa protein with an estimated molecular weight of 24.4 kDa (24K protein). The sequences of the 22K and 24K proteins encoded by the 3′ proximal ORFs 4 and 5, respectively, did not exhibit significant similarities to the proteins in the GenBank databases. 

To confirm the presence of BcTLV in the original psyllid sample, PSW, and to check for its presence in other psyllid and tomato samples collected, three primer pairs were designed (Supplementary Table S1) to produce DNA fragments ranging between 421 and 702 bp and spread along the BcTLV genome (see Figure 1a). In the end-point RT-PCR tests, the expected PCR fragments were amplified when the original RNA sample used for the HTS experiments was tested (Figure 1b); these amplified PCR fragments were subjected to Sanger sequencing and confirmed to represent BcTLV sequences, being 100% identical to the HTS-derived nucleotide sequence. RNA was also extracted in April 2022 from two tomato plants kept in the cage where the PSW colony was maintained and was subjected to RT-PCR testing using the same three primer pairs but was found to be negative for BcTLV (Figure 1b). On the other hand, the PSW colony psyllids collected in April 2022 from these tomato plants maintained in Wapato, WA, were found to be BcTLV-positive. 

### 3.2. Genome Analysis and Confirmation of the Presence of New Solemo-like Viruses in Psyllids

**Figure 2:**
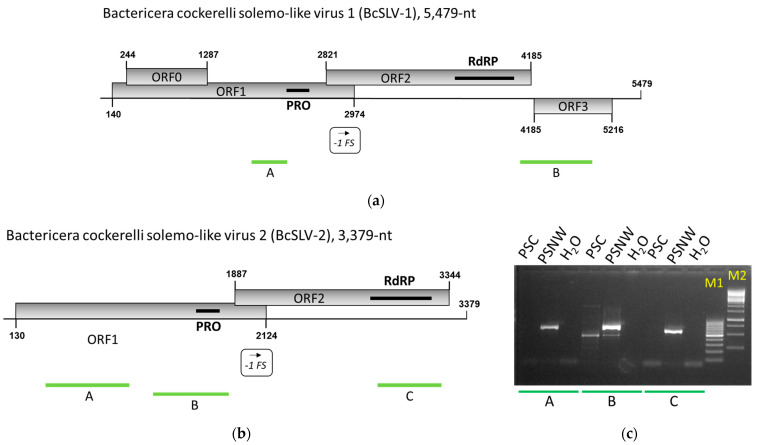
Organization of the genomes of Bactericera cockerelli solemo-like virus 1 (BcSLV-1) (**a**) and of Bactericera cockerelli solemo-like virus 2 (BcSLV-2) (**b**), and the laboratory detection of BcSLV-2 in insects collected from the laboratory colony (**c**). (**a**) Schematic diagram of the BcSLV-1 genome with positions of the four ORFs coding for the 38K protein (ORF0), 104K protein (ORF1), 50K protein (ORF2), and 37.7K protein (ORF3) shown as lightly shaded rectangles, with nucleotide numbers for the start and stop codons indicated. Black solid rectangles indicate positions of the known protein domains identified in the 104K and the 50K protein: protease (PRO) and RNA-dependent RNA polymerase (RdRP). The green bars underneath the BcSLV-1 genome represent the location of the RT-PCR fragments amplified by the two primer pairs for testing (A, BcSLV1_F1/R1, 467-bp; B, BcSLV1_F2/R2, 975-bp; Supplementary Table S1). (**b**) Schematic diagram of the BcSLV-2 genome with positions of the two ORFs coding for the 73K protein (ORF1) and 53K protein (ORF2) shown as lightly shaded rectangles, with nucleotide numbers for the start and stop codons indicated. Black solid rectangles indicate positions of the known protein domains identified in the 73K and the 53K protein: protease (PRO) and RNA-dependent RNA polymerase (RdRP). The green bars underneath the BcSLV-2 genome represent the location of the RT-PCR fragments amplified by the three primer pairs for testing (A, BcSLV2_F1/R1, 766-bp; B, BcSLV2_F2/R2, 760-bp; C, BcSLV2_F3/R3, 581-bp; Supplementary Table S1). The sequences of the primers used for PCR amplifications are listed in Supplemental Table S1. (**c**) Testing for BcSLV-2 by endpoint RT-PCR in the original *B. cockerelli* insects collected from the Wapato, WA, psyllid colonies; three sets of primers (see (**b**); Supplementary Table S1) were used for the testing here, producing a 766 bp PCR product (A), a 760 bp product (B), and a 581 bp product (C); two colonies, PSC and PSNW, were sampled; (H_2_O) negative non-template control. M1 is a marker lane, loaded with the 100 bp DNA ladder; M2 is the 1000 bp ladder.

Four additional contigs ranging from 3365 to 5479 nt were revealed in the PSNW and KPF samples which encoded two (three contigs) or four (one contig) ORFs exceeding 100 codons. A BLASTn search through the GenBank database returned no similar nucleotide sequences, while the CD-Search program [46] identified the RdRP and PRO domains of solemo-like viruses in two of the protein products encoded by ORFs 1 and 2 of these four contigs. Two of the smaller contigs found in the KPF (3379 nt; 45,607 reads mapped) and PSNW (3498 nt; 33,298 reads mapped) samples displayed 99.7% identity at the nucleotide level, while the third contig found in the PSNW (3365 nt; 52,781 reads mapped) sample was 87% identical at the nucleotide sequence level to both the 3379 nt and 3498 nt contigs. Given the presence of the RdRP domain in the encoded proteins, these four contigs were assumed to represent complete or nearly complete genomes of two novel positive-strand RNA viruses named “Bactericera cockerelli solemo-like virus 1” (BcSLV-1) and “Bactericera cockerelli solemo-like virus 2” (BcSLV-2) (Figure 2). The PSNW sample apparently contained two distinct genetic variants of BcSLV-2, which were designated BcSLV-2_1 and BcSLV-2_2 (Table 1). These nearly complete genomes were subjected to a more thorough molecular and bioinformatics analysis. The sequences have been deposited into GenBank under the accession numbers PP408655 (BcSLV-1), PP408657 (BcSLV-2_1), and PP408658 (BcSLV-2_2).

The sequenced genome of BcSLV-1 (5479 nt; 240 reads mapped) was conceptually translated and found to encode four ORFs (Figure 2a). The largest, ORF1, spans positions 140 to 2974, encoding a 104 kDa protein product (104K protein) containing a PRO domain with presumed protease activity. The downstream ORF2 overlaps ORF1 and spans positions 2821 to 4185, encoding a 50 kDa protein product (50K protein), which is likely expressed through a −1 translational frameshift. This 50K protein was found to contain an easily identifiable RdRP domain exhibiting significant similarities with the RdRPs of solemo- and solemo-like viruses when subjected to the tBLASTn search through the GenBank databases. The smaller ORF0 completely overlaps with the 104K protein ORF1, starting at position 244 and ending at position 1287, encoding a 38 kDa protein (38K protein). This 38K protein seems to resemble similarly encoded products in the genomes of some other solemoviruses infecting plants that are required for virus spread in a plant host, and also function as movement proteins and gene silencing suppressors [49,50]; the sequence of this 38K protein did not exhibit significant similarities to the proteins in the GenBank databases though. The 3′ proximal ORF3 starts at position 4185 and ends at position 5216, encoding a 343 aa protein with an estimated molecular weight of 37.7 kDa (37.7K protein). This ORF3-encoded, 37.7K protein did not exhibit significant similarity with other proteins in the GenBank databases when subjected to a search using the tBLASTn program. 

The sequenced genome of BcSLV-2 was conceptually translated and found to encode two ORFs (Figure 2b). The largest ORF1 spans positions 130 to 2124, encoding a 73 kDa protein product (73K protein) containing a PRO domain with presumed serine protease activity. The downstream ORF2 overlaps ORF1 and spans positions 1887 to 3344, encoding a 53 kDa protein product (53K protein), which is likely expressed through a −1 translational frameshift. This 53K protein contains an easily identifiable RdRP domain exhibiting significant similarities with the RdRPs of solemo- and solemo-like viruses when subjected to a tBLASTn search through the GenBank databases. 

To confirm the presence of the BcSLV-1 and BcSLV-2 sequences in the original psyllid samples, KPF and PSNW, and to check for its presence in the other psyllid samples collected, two (BcSLV-1) and three (BcSLV-2) primer pairs were designed (Supplementary Table S1) to produce RT-PCR amplified DNA fragments ranging between 467 and 975 nt and spread along the BcSLV-1 and BcSLV-2 genomes (see Figure 2a,b). In the end-point RT-PCR tests with the BcSLV-2 primers, the expected PCR fragments were amplified when the original PSNW RNA sample used for the HTS experiments was tested (Figure 2c); these amplified PCR fragments were subjected to Sanger sequencing and confirmed to represent BcSLV-2 sequences, 100% identical to the HTS-derived nucleotide sequence. The presence of the BcSLV-1 sequences in the original KPF sample collected from the field could not be confirmed by RT-PCR possibly due to the low number of virus copies in the original KPF sample; only 240 reads were mapped to the BcSLV-1 genome in this sample.

### 3.3. Phylogenetic Analyses and Taxonomy of BcTLV, BcSLV-1, and BcSLV-2

**Figure 3:**
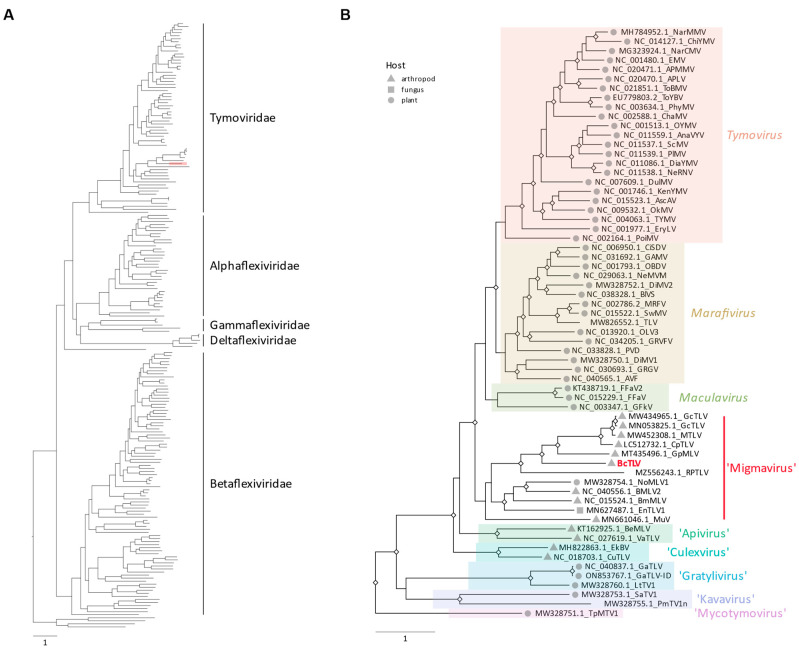
Phylogenetic analysis of the replicase proteins of members of the order *Tymovirales*. (**A**) A maximum likelihood phylogenetic tree was inferred from the alignment of the replicase amino acid sequences from representative viruses of the *Tymovirales* order, using IQtree2 with ModelFinder to choose the best fit model and branch support estimated using UltraFast bootstrapping (Ufboostrap) and SH-like approximate ratio test (SH-aLRT). The branch for BcTLV is highlighted in light red. The tree was rooted using an outgroup comprising replicase domains of viruses from the *Martellivirales* and *Hepellivirales* orders, with the root placed on the edge connecting the ingroup and outgroup. (**B**) A close-up from the tree in A on the Tymoviridae family shows the placement of the BcTLV sequence in this clade. Significant bootstrap support, as estimated by SH-aLRT and Ufbootstrap values, is indicated by a diamond shape at the concerned internal nodes (SH-aLRT > 80% and Ufbootstrap > 95%). When known, the host in which each virus represented in the tree had been initially identified, is indicated by a grey symbol at the corresponding branch tip (except viruses in the outgroup). The original, fully annotated tree is available in the Supplemental Information (Supplementary Figure S1). The scale bar shows the number of substitutions per site.

The number and relative positions of the ORFs encoded by BcTLV resembled the genome organizations of tymo- and tymo-like viruses (Figure 1a). In particular, the genome size of BcTLV, the number of the encoded ORFs and their overall arrangement, in addition to the sequence similarity in the RdRP domain, resembled the grapevine fleck virus (GFkV) genome, a phloem-limited maculavirus [51,52,53]. In the pair-wise comparisons, however, BcTLV and GFkV exhibited distant homology for only two of the encoded protein products, replication-associated polyprotein (190K protein, 38% identity) and CP (30% identity); no significant similarity was found between other protein products encoded by the BcTLV and GFkV genomes. In the phylogenetic tree of the replicase proteins of viruses belonging to the order *Tymovirales* (Supplementary Figure S1; Figure 3A), BcTLV was placed among various tymo-like viruses found in insect and plant hosts in a sister clade to the genera *Tymovirus*, *Marafivirus*, and *Maculavirus* (Figure 3B). The phylogeny of the CP (Supplementary Figure S2) appeared consistent with the replicase phylogeny in the placement of the BcTLV CP among the divergent group of tymo-like viruses found in insect and plant hosts in a sister clade to the genus *Maculavirus*. The deep and distinct separation of the BcTLV branch from other tymo-like viruses in this diverse clade (Figure 3B) may suggest the possibility that BcTLV represents a new taxon, but this cannot be discussed until more virus sequences similar to BcTLV are found. 

**Figure 4:**
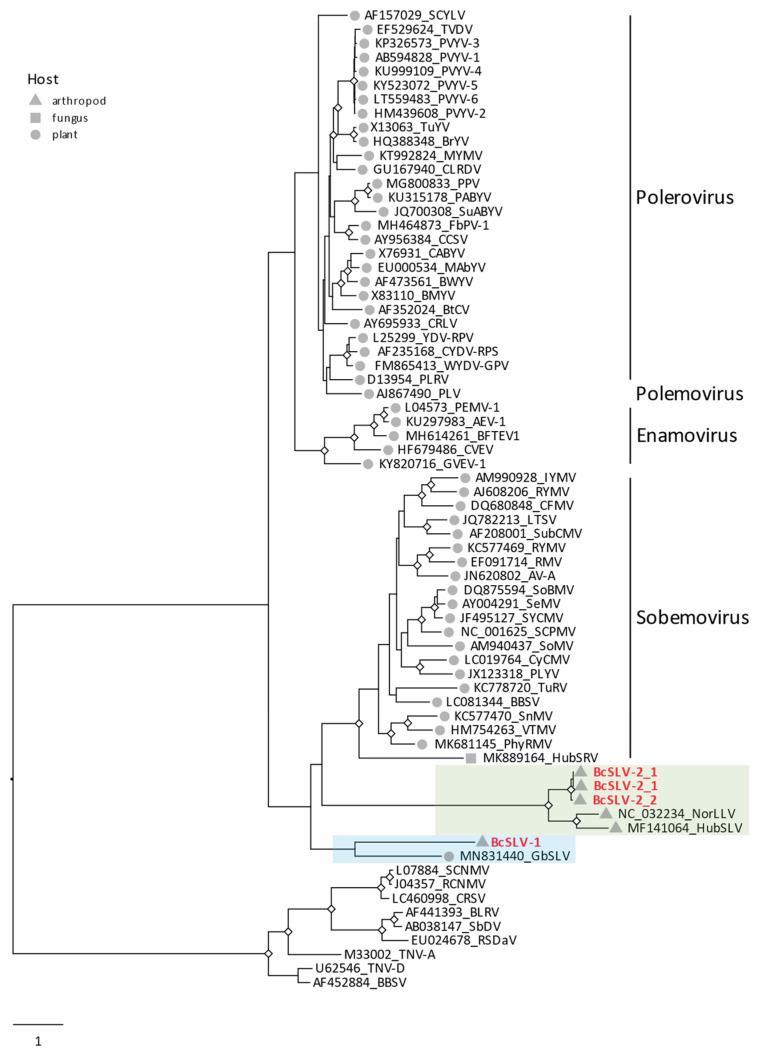
Phylogenetic analysis of the RdRP domains of members of the family *Solemoviridae*. Predicted RdRP domains for BcSLV-1, BcSLV-2_1, and BcSLV-2_2 were added to the solemovirus RdRP alignment provided by ICTV along with the following sequences: MF141064 (Norway luteo-like virus 1), NC_032234 (Hubei sobemo-like virus 29), MK889164 (Hubei sobemo-like virus 1), MN831440 (Gingko biloba sobemo-like virus). A maximum likelihood phylogenetic tree was inferred from the alignment using IQtree2 with ModelFinder to choose the best fit model, and branch support was estimated using UltraFast bootstrapping (UFboostrap) and SH-like approximate ratio test (SH-aLRT). Significant bootstrap support, as estimated by SH-aLRT and UFbootstrap values, is indicated by a diamond shape at the concerned internal nodes (SH-aLRT > 80% and UFbootstrap > 95%). The tree was rooted using an outgroup comprising RdRP domains of viruses from the *Tombusviridae* family, with the root placed on the edge connecting the ingroup and outgroup. The clades comprising the BcSLV RdRP sequences are highlighted in light green (BcSLV-1) and light red (BcSLV-2_1 and BcSLV2_2). When known, the host in which each virus represented in the tree had been initially identified, is indicated by a grey symbol at the corresponding branch tip (except viruses in the outgroup). The scale bar indicates the number of substitutions per site.

The organization of the BcSLV-1 and BcSLV-2 genomes coding for four and two ORFs, respectively, encoding the serine protease and RdRP domains with two overlapping ORFs expressed via a putative translational frameshift in both genomes, resembled viruses from the family *Solemoviridae*. Indeed, in the BLASTx searches, the protein product encoded by ORF 2 of BcSLV-1 exhibited the closest affinity to the RdRP of Ginkgo biloba sobemo-like virus [54], with a 38% identity with the ORF2-encoded protein. No other hits for the ORF0-, ORF1-, and ORF3-encoded proteins were detected in the BLASTx searched through the GenBank database. In similar BLASTx searches, protein products encoded by ORFs 1 and 2 of BcSLV-2 exhibited the closest affinity to the similarly encoded proteins of Norway luteo-like virus 1 [27], with 33% identity with the ORF1-encoded protein and 55% identity with the ORF2-encoded protein (84% coverage). In the phylogenetic tree of the RdRPs of viruses belonging to the family *Solemoviridae* (Figure 4), BcSLV-1 and BcSLV-2 were placed inside the diverse lineage of various solemo-like viruses found mainly in plant hosts in two sister clades to the genus *Sobemovirus* (Figure 4). The BcSLV-1 RdRP was grouped in the same lineage with Ginkgo biloba sobemo-like virus (35.9% in pairwise aa identity) while the BcSLV-2 RdRP sequences were placed in a separate lineage close to two arthropod viruses, Hubei sobemo-like virus 29 (53.4% aa identity) and Norway luteo-like virus 1 (51.5% aa identity). The deep and distinct separation of both the BcSLV-1 and BcSLV-2 branches from other sobemoviruses in this diverse clade (Figure 4) may suggest the possibility that BcSLV-1 and BcSLV-2 represent new taxa, but this may depend on more virus sequences similar to BcSLV-1 and BcSLV-2 being found.

### 3.4. BcPLV, BcTLV, and BcSLV-1 and -2 Can Be Found in Field-Collected Psyllids

In the samples from 2023, most of the 218 field-collected psyllids were found to be Lso-negative. These same 218 psyllids were tested for the presence of BcPLV, BcTLV, BcSLV-1, and BcSLV-2 using RT-PCR, as described in the Materials and Methods (Table 2). BcPLV was found to be quite common in the field-collected psyllids, present in about 27% of all the tested psyllids collected between July and September of 2023 (see Table 2). Four BcTLV-positives were identified based on the size of the PCR band amplified with PSW_F3/R3 (Table 2). One of the insects, EJW19668, collected on August 18, 2023, in Canyon County (Idaho), produced a prominent PCR band of 516 bp (Figure 1c) when tested with primers PSW_F3/R3 (see Figure 1c). This PCR product was submitted for Sanger sequencing and its nucleotide sequence was found to be 99.0% identical to the HTS-derived BcTLV sequence for the BcTLV isolate from the PSW psyllid colony maintained in Wapato, WA. Apparently, BcTLV circulated among the field potato psyllids, although its prevalence was not very high, staying below 2%. BcSLV-1 was found only in five psyllids during the season (4.1%), while BcSLV-2 was apparently the most prevalent in the field psyllids, found in over 50% of the tested samples (Table 2).

## 4. Discussion

*B. cockerelli* is a phloem feeder from the family *Triozidae* which, together with the families *Liviidae*, and *Psyllidae*, belongs to the superfamily Psylloidea, the jumping plant lice [55,56]. Some of the psyllid species from this superfamily are agricultural pests, affecting important crops, like citrus and potato, either directly or by vectoring bacterial pathogens associated with devastating diseases such as huanglongbing and zebra chip [7,9]. The management of psyllid-vectored pathogens is heavily reliant on insecticides, which is not sustainable from the standpoints of insecticide resistance, nontarget effects on natural enemies, and regulations that will limit the use of such tools [7,12]. One alternative to insecticides may be the use of insect viruses [12,15,57]. However, despite the wide use of HTS methodologies, the knowledge of psyllid viruses has lagged relative to other insect families, with only a limited number of viruses reported in psyllids [58]. The presence of insect viruses was established a few years ago in the Asian citrus psyllid, where several viruses from various families were found, representing picorna-like, flavi-like, and reo-like viruses [12,13,14,15]. A single picorna-like virus was found in potato/tomato psyllids [8] and another picorna-like virus in wild lime psyllids (LfPLV; [18]). Hence the data obtained in this study quadrupled the number of viruses found in *B. cockerelli,* which now stands at four: BcPLV, BcTLV, BcSLV-1, and BcSLV-2 (Table 1). 

The previously found BcPLV belongs to the family *Iflaviridae* [8] and is closely related to two other picorna-like viruses found in the Asian citrus psyllid (DcPLV; [13]) and in wild lime psyllids (LfPLV; [18]). All three displayed the same genome organization and a very similar domain architecture of their polyproteins. However, in RdRP phylogeny [18], picorna-like viruses from whitefly *Bemisia tabaci* (BtPLV-1; [59]) and crab spider *Diaea subdola* (FIf-2; MZ210044) were also placed in the same RdRP clade, while the genome organization and domain architecture within the polyprotein of BtPLV and FIf-2 differed from those of the psyllid picorna-like viruses. Apparently, the refined classification of these five picorna-like viruses awaits additional sequences from other related arthropod viruses. 

The order *Tymovirales*, comprising five ICTV-approved families including the family *Tymoviridae*, is classified in the so-called ‘Branch 3’ of positive-strand RNA viruses, and placed as a sister clade to alpha-like viruses [60,61]. Three of the genera of the family, *Tymoviridae*, *Tymovirus*, *Marafivirus*, and *Maculavirus*, comprise plant viruses [62], while a number of tymovirids were described recently from arthropod and fungal hosts [20]. Hence, the discovery of BcTLV, a tymo-like virus, in a psyllid host (Table 1; Figure 1a–c) does not seem unusual. In phylogenetic trees inferred for the replicase and CP domains of a large number of tymovirids (Figure 3A,B; Supplementary Figure S2), BcTLV was placed into a diverse lineage which includes virus sequences from plant and non-plant hosts; this lineage was placed as a sister clade to the plant-infecting genera *Tymovirus*, *Marafivirus*, and *Maculavirus*, both for replicase and CP proteins (Figure 3A,B; Supplementary Figure S2). The BcTLV sequences were found to be the closest to a group of tymovirids from insect hosts, such as mosquitoes and beetles; we propose that this diverse lineage be named ‘Migmavirus’ after the Greek word migma (μίγμα, mixture). 

The family *Solemoviridae* belongs to the so-called ‘Branch 2’ of positive-strand RNA viruses comprising the picorna-like virus supergroup [60,61]. The great majority of the virus sequences currently classified into the family *Solemoviridae* are of plant origin [26], with only two reports of solemo-like viruses from arthropods [27,28]. The two solemo-like viruses reported here, BcSLV-1 and BcSLV-2, add two additional arthropod virus sequences to *Solemoviridae*. Perhaps unsurprisingly, one of the discovered solemo-like viruses, BcSLV-2, was phylogenetically placed very close to these two previously reported arthropod viruses, forming a tight clade separate from all other clades of *Solemoviridae*, separate from the well-defined and ICTV-approved genera *Sobemovirus*, *Enamovirus*, and *Polerovirus* (Figure 4). This tight clade comprising related viruses from such arthropods as psyllids, dragon flies, and ticks may represent a new taxon at the genus level within the family *Solemoviridae,* which we propose to name Ixorivirus. BcSLV-1, on the other hand, was grouped with the Gingko biloba sobemo-like virus 1 (GbSLV-1), a plant virus (Figure 4). In this case, for the phylogenetic placement of BcSLV-1, the different type of host (insect vs. plant) did not seem to be important, indicating that additional related sequences may be needed for the accurate classification of both BcSLV-1 and GbSLV-1. 

The current study is the first evidence that all four psyllid viruses identified previously in potato psyllid colonies, BcPLV [8], or in this work, i.e., BcTLV, BcSLV-1, and BcSLV-2 (Table 1), circulate in psyllid populations in potato fields (see Table 2). The dynamics of the psyllid virus prevalence that can be found in insects during a growing season needs to be studied for a longer period to determine if changes in psyllid numbers in the field [29,30,31,32,33] may correlate (or not) with the presence (and prevalence) of individual or mixed virus infections in the field. Numerically, based on this single-season study, the prevalence of all psyllid viruses seemed to increase by the end of the season (Table 2). However, since this is only a single-season dataset, no conclusions can be made about the potential role of these four psyllid viruses in controlling psyllid numbers in potato fields, and any possible role of these viruses in controlling populations of the field psyllids remains to be elucidated. 

## 5. Conclusions

Three new positive-strand RNA viruses were found in potato/tomato psyllid populations from laboratory colonies and collected from potato fields. One new virus, BcTLV, belonged to the family *Tymoviridae*, and two new viruses, BcSLV-1 and BcSLV-2, belonged to the family *Solemoviridae*. All three new viruses, BcTLV, BcSLV-1, and BcSLV-2, along with the previously reported BcPLV, were discovered to be circulating among potato/tomato psyllids collected from potato fields in southern Idaho during the 2023 summer season. 

## Figures and Tables

**Table 1:** Summary of the potato psyllid samples subjected to the high-throughput sequencing (HTS) and virus-specific sequences found in the HTS outputs. Bold italic font designates new virus sequences discovered.

Psyllid Sample ID ^a^	Origin	Date Collected	Host Plant	Virus Sequences Found ^b^	Largest Contig Size, nt	GenBank Accession Number
PSC	Wapato, WA	Feb-2021	potato	PVS	8546	PP408647
				BcPLV	10,122	PP408652
PSW	Wapato, WA	Feb 2021	potato/ tomato	***BcTLV***	***6843***	PP408654
				PVS	4339	PP408649
				PVY	3729	PP408651
				BcPLV	10,150	PP408653
PSNW	Wapato, WA	Feb 2021	potato	PVS	8537	PP408650
				***BcSLV-2_1***	***3498***	PP408657
				***BcSLV-2_2***	***3365***	PP408658
KPF	Kimberly, ID	Aug 2020	potato	PVS	8407	PP408648
				***BcSLV-1***	***5479***	PP408655
				***BcSLV-2_1***	***3379***	PP408656

^a^ PSC = psyllid colony, central haplotype; PSW = psyllid colony, western haplotype; PSNW = psyllid colony, northwestern haplotype; KPF = Kimberly field psyllids. ^b^ Virus abbreviations: PVS = potato virus S; PVY = potato virus Y; BcPLV = Bactericera cockerelli picorna-like virus; BcTLV = Bactericera cockerelli tymo-like virus; BcSLV-1 = Bactericera cockerelli solemo-like virus 1; BcSLV-2 = Bactericera cockerelli solemo-like virus 2; numbers after a dash in BcSLV-2 designate genetic variants of the virus.

**Table 2:** RT-PCR testing for psyllid viruses in field psyllids captured in southern Idaho during the 2023 season of psyllid monitoring. Total RNA was extracted from individual psyllids and subjected to RT-PCR using BcPLV-specific primers described in [8] or with specific primers listed in Supplementary Table S1. Numerator, number of virus-positive psyllids; denominator, number of psyllids tested.

Date of Collection	BcPLV ^a^	BcTLV ^a^	BcSLV-1 ^a^	BcSLV-2 ^a^
7 July	0/2	0/2	0/2	0/2
11 July	1/3	0/3	2/3	1/3
14 July	2/14	0/14	0/14	4/14
21 July	8/10	0/10	0/10	6/10
14 August	0/29	0/29	0/29	5/29
11 August	0/23	1/23	0/23	12/23
15 August	0/7	0/7	0/7	2/7
18 August	9/44	1/44	5/44	29/44
25 August	5/25	0/25	1/25	17/25
28 August	21/28	0/28	1/28	19/28
1 September	9/15	0/15	0/15	7/15
8 September	1/6	2/6	0/6	3/6
14 September	2/12	0/12	0/12	5/12
**Total:**	58/218	4/218	9/218	110/218

^a^ Virus abbreviations: BcPLV = Bactericera cockerelli picorna-like virus; BcTLV = Bactericera cockerelli tymo-like virus; BcSLV-1 = Bactericera cockerelli solemo-like virus 1; BcSLV-2 = Bactericera cockerelli solemo-like virus 2.

## Data Availability

The genome sequences reported are available in GenBank under accession numbers PP408647-PP408658 (see Table 1). The raw sequence data were deposited in the NCBI Sequence Read Archive (SRA) under BioProject accession number PRJNA1080349.

## Supplementary Materials

The following supporting information can be downloaded at https://www.mdpi.com/article/10.3390/v16030415/s1, Figure S1: Full phylogenetic tree of the replicase proteins of *Tymovirales* order members; Figure S2: Phylogenetic analysis of the coat protein of *Tymoviridae* family members; Table S1: Primers used to confirm and validate the presence of three psyllid viruses in the colony and field psyllids.
