# Supplementary material for: Bactericera cockerelli Picorna-like Virus and Three New Viruses Found Circulating in Populations of Potato/Tomato Psyllids (Bactericera cockerelli)
Source: Viruses. 2024 Mar 8;16(3):415. doi: 10.3390/v16030415 (PMC10975263; doi:10.3390/v16030415)
Supplement: Supplementary file 1 [file viruses-16-00415-s001.zip › viruses-2879043-supplementary.pdf]

Supplemental Table S1. List of primer pairs used in this study. The sequences for all primer pairs used for RT-PCR testing of each virus (BcTLV, BcSLV-1 and BcSLV-2) are given in the table, with start and end positions based on the corresponding contig assembled from HTS data as described in main text, Tm for each primer as calculated by Primer3 implemented in Geneious Prime with default parameters, and length of predicted PCR product.

Name	Start	End	Sequence	Tm (°C)	Length (bp)
**BcTLV_A_F**	982	1001	TTCGAACTCTTCGCGTCACA	60.0	702
**BcTLV_A_R**	1664	1683	CACACTCGGGTTCCGAAGAA	60.0	
**BcTLV_B_F**	4507	4526	CCGAGCATCTCATCCAGGTC	60.0	421
**BcTLV_B_R**	4908	4927	GAATGGCCGACGCTGAATTC	60.0	
**BcTLV_C_F**	6171	6190	CCTCGGAGGAAGTCCCTACA	60.0	516
**BcTLV_C_R**	6667	6686	CGTGTTTGTACGCGGGTTTT	60.0	
**BcSLV1_A_F**	1573	1592	GCCCTTCAAGTGCCAACTTG	60.0	467
**BcSLV1_A_R**	2020	2039	CGTGCTTTCGATAACGGCTG	60.0	
**BcSLV1_B_F**	4023	4042	CGAGGCTCGTATGGAGAACC	60	975
**BcSLV1_B_R**	4978	4997	TATGTTGCCCCCATAGGGGA	60.0	
**BcSLV2_A_F**	274	293	AATGGGCAGCATTGTTTGGG	59.7	766
**BcSLV2_A_R**	1020	1039	TGCCTGAAACCATACCCCAC	60.0	
**BcSLV2_B_F**	1189	1208	AGACAACGTGGACCAAGCTC	60.3	760
**BcSLV2_B_R**	1929	1948	CAGCCGTACTCAACAGGAGG	60.1	
**BcSLV2_C_F**	2849	2868	GGCTGGGATTGGACAGTACA	59.4	581
**BcSLV2_C_R**	3407	3429	GTGAGTTTTGCTCCCATCTTAGC	59.9	

Supplemental Figure S1

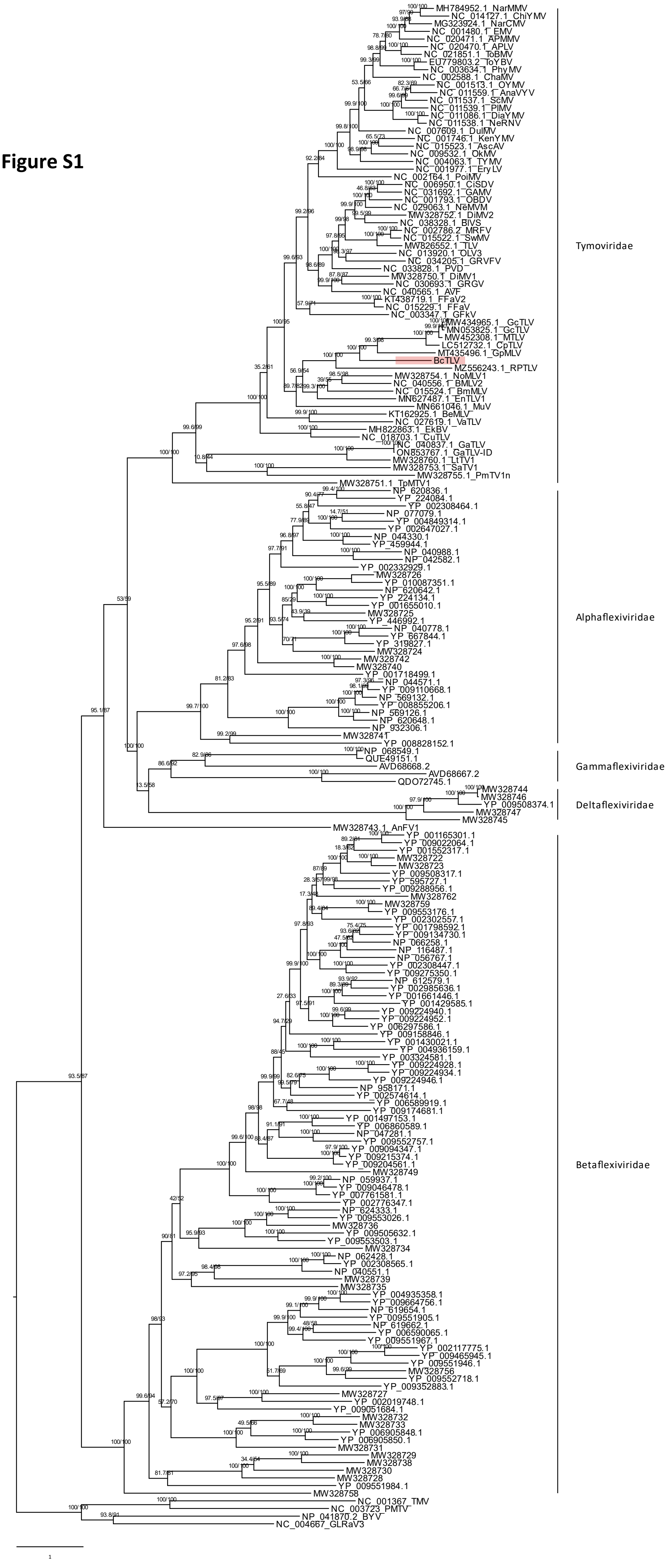

Supplemental Figure S1. Phylogenetic analysis of the replicase proteins of members of the order *Tymovirales*. maximum-likelihood phylogenetic tree was inferred from the alignment of the replicase aminoacid sequences from representative viruses of the *Tymovirales* order, using IQtree2 with ModelFinder to choose the best fit model and branch support estimated using SH-like approximate-ratio test (SH-aLRT) and UltraFast bootstrapping (UFbootstrap), with 1000 replicates. Both bootstrap values are indicated at the nodes (SH-aLRT/UFbootstrap). The branch for BcTLV is highlighted in light red. The tree was rooted using an ougroup comprising replicase domains of viruses from the *Martellivirales* and *Hepellivirales* orders, with the root placed on the edge connecting the ingroup and outgroup. The scale bar shows the number of substitutions per site.

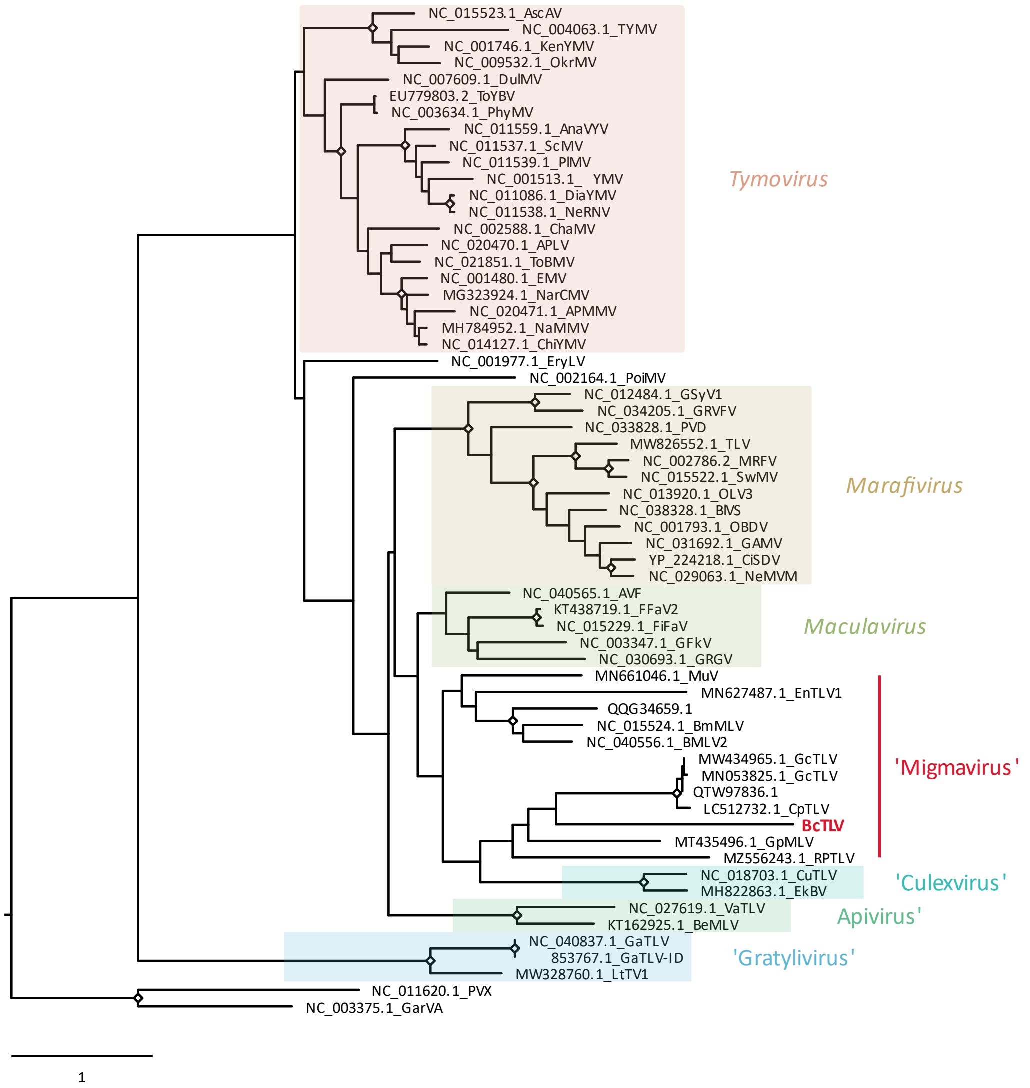

Supplemental Figure S2. Phylogenetic analysis of the coat proteins of members of the family *Tymoviridae*. maximum-likelihood phylogenetic tree was constructed from the alignment of the coat protein aminoacid sequences from representative viruses of the *Tymoviridae* family, using IQtree2 with ModelFinder to choose the best fit model, and branch support estimated using UltraFast bootstrapping (UFbootstrap) and SH-like approximate-ratio test (SH-aLRT). The tree was rooted using outgroup sequences of coat proteins of viruses from the *Alphaflexiviridae* family, with the root placed on the edge connecting the ingroup and outgroup. Significant bootstrap support, as estimated by SH-aLRT and UFbootstrap values, is indicated by a diamond shape at the concerned internal nodes (SH- LRT > 0% and UFbootstrap > %). The scale bar shows the number of substitutions per site.
